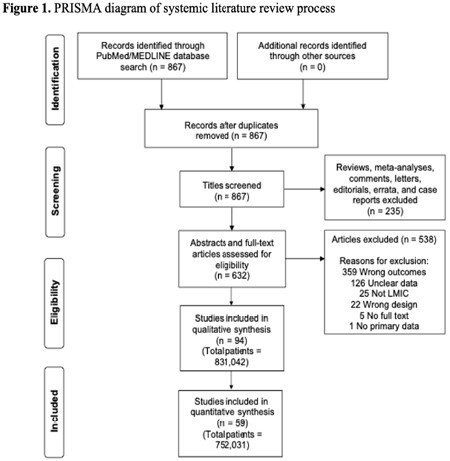# 571 The Trauma Burden of Pediatric Burns in Low- and Middle-Income Countries: A Systematic Review

**DOI:** 10.1093/jbcr/irae036.205

**Published:** 2024-04-17

**Authors:** Flora Park, Andres Ruiz, Jonathan Pang, Kirsten Young, Bradley Roth, Emily Smith, Theresa L Chin

**Affiliations:** Department of Surgery, Division of Trauma, Burns and Surgical Critical Care, University of California, Irvine School of Medicine, Orange, CA; Department of Surgery, Huntington Health, Pasadena, CA; Duke Global Health Institute, Durham, NC; Department of Surgery, Division of Trauma, Burns and Surgical Critical Care, University of California, Irvine School of Medicine, Orange, CA; Department of Surgery, Huntington Health, Pasadena, CA; Duke Global Health Institute, Durham, NC; Department of Surgery, Division of Trauma, Burns and Surgical Critical Care, University of California, Irvine School of Medicine, Orange, CA; Department of Surgery, Huntington Health, Pasadena, CA; Duke Global Health Institute, Durham, NC; Department of Surgery, Division of Trauma, Burns and Surgical Critical Care, University of California, Irvine School of Medicine, Orange, CA; Department of Surgery, Huntington Health, Pasadena, CA; Duke Global Health Institute, Durham, NC; Department of Surgery, Division of Trauma, Burns and Surgical Critical Care, University of California, Irvine School of Medicine, Orange, CA; Department of Surgery, Huntington Health, Pasadena, CA; Duke Global Health Institute, Durham, NC; Department of Surgery, Division of Trauma, Burns and Surgical Critical Care, University of California, Irvine School of Medicine, Orange, CA; Department of Surgery, Huntington Health, Pasadena, CA; Duke Global Health Institute, Durham, NC; Department of Surgery, Division of Trauma, Burns and Surgical Critical Care, University of California, Irvine School of Medicine, Orange, CA; Department of Surgery, Huntington Health, Pasadena, CA; Duke Global Health Institute, Durham, NC

## Abstract

**Introduction:**

Pediatric burns place a disproportionate burden of injury on low- and middle-income countries (LMICs). With this systematic review, we aimed to illustrate trends in pediatric burn epidemiology in LMICs and describe possible areas for international intervention.

**Methods:**

We performed a systematic review of existing literature on pediatric burns in LMICs. PubMed and MEDLINE databases were searched for primary studies conducted in LMICs reporting pediatric burn incidence, male-to-female ratio, mortality, and mechanism of injury. A quality assessment analysis was performed using the Methodological Index for Non-Randomized Studies (MINORS) criteria.

**Results:**

The search yielded 867 unique articles, of which 14 studies met MINORS criteria. The included studies covered pediatric burn data from 9 LMICs: Rwanda, Malawi, Uganda, Bangladesh, Sudan, Pakistan, Cameroon, Kenya, and Fiji. Upon qualitative analysis, burns were found to be the most common mechanism of injury and cause of death compared to other traumatic injuries in children under 5-years-old. The most common mechanism of burn was reported to be scald injury due to cooking accidents. Incidence of pediatric burn injury was difficult to calculate for LMICs as only three studies reported this information. The incidence of pediatric burns per 100,000 in Sudan, Kenya, and Fiji respectively were reported to be 1010, 4887, and 32.6. Lower socioeconomic status and access to surgery were cited as the most common barriers to adequate burn care in LMICs.

**Conclusions:**

The burden of pediatric burn injury on LMICs is significant and can be characterized using a quality-controlled systematic review. Further standardized studies must be conducted in collaboration with LMICs on this topic.

**Applicability of Research to Practice:**

Our study illuminates potential routes for improvement in pediatric burn management and prevention in LMICs, and we hope to encourage more conversation around this topic led by LMIC researchers and healthcare providers.